# Dietary Replacement Effect of Fish Meal by Tuna By-Product Meal on Growth and Feed Availability of Red Sea Bream (*Pagrus major*)

**DOI:** 10.3390/ani14050688

**Published:** 2024-02-22

**Authors:** Seong Il Baek, Sung Hwoan Cho

**Affiliations:** 1 Department of Convergence Study on the Ocean Science and Technology, Korea Maritime and Ocean University, Busan 49112, Republic of Korea; qortjddlff@naver.com; 2 Division of Convergence on Marine Science, Korea Maritime and Ocean University, Busan 49112, Republic of Korea

**Keywords:** *Pagrus major*, replacement of fish meal, polynomial orthogonal contrast, regression analysis, economic profit index

## Abstract

**Simple Summary:**

Fish meal is widely used as a feed ingredient in formulated feeds for marine fish species due to its high nutritional value and palatability. However, the increasing cost and limited availability of fish meal highlight the high need to look for an alternative protein source for fish meal in fish feeds to achieve sustainable aquaculture. Tuna by-product meal, derived from the tuna canning process, shows promise as a viable substitute for fish meal in fish feeds. This study aimed to investigate the effect of replacing fish meal with tuna by-product meal on the growth of red sea bream. The findings of this study suggested that 40% fish meal replacement with tuna by-product meal is viable without compromising growth, feed consumption, and feed utilization, while simultaneously providing the highest economic return for fish farmers.

**Abstract:**

The effect of substituting fish meal (FM) by tuna by-product meal (TBM) on growth and feed availability of red sea bream (*Pagrus major*) was investigated. Six experimental diets were crested to be isonitrogenous (51.5%) and isolipidic (14.5%). The control (Con) diet contained 55% FM. FM substitution in the Con diet was made in increments of 20 percentage points (20, 40, 60, 80, and 100%), named as the TBM20, TBM40, TBM60, TBM80, and TBM100 diets, respectively. Juvenile red sea bream were stocked into 18, 300 L flow-through tanks (50 fish/tank). Red sea bream were hand-fed with each diet until satiation for 8 weeks. No statistical differences in weight gain, specific growth rate (SGR), and feed consumption were found among red sea bream fed the Con, TBM20, and TBM40 diets. Furthermore, feed utilization of fish fed the TBM20, TBM40, TBM60, and TBM80 diets was comparable to red sea bream fed the Con diet. The biological indices, biochemical composition, and hematological parameters of fish were not statistically altered by dietary FM replacement with TBM. The greatest economic profit index was achieved in the TBM40 diet. In conclusion, the replacement of 40% FM with TBM in red sea bream diet appears to be the most recommendable approach without producing retarded growth and feed availability, but maximizing EPI to farmers.

## 1. Introduction

Red sea bream (*P. major*) is a representative fish species commonly farmed in Eastern Asia, including the Republic of Korea (hereafter, Republic of Korea) and Japan. The annual aquaculture production of red sea bream in Korea has continuously elevated from 2755 metric tons in 2013 to 8313 metric tons in 2021 [1]. Carnivorous fish species typically demand animal-origin high protein levels in their feeds, and the quality of fish feed is largely contingent on their protein sources, constituting two-thirds of the total feed cost [2]. Fish meal (FM) remains a primary and costly protein source in formulated fish diets, due to its high nutritional value and excellent palatability [3,4]. Fish feeds must contain a higher proportion of FM compared to feeds for terrestrial livestock to fulfill the nutritional requirements [5]. Nevertheless, the global production rate of FM has decreased by an average of 1.7% per annum since 1995, due to the regulation of fisheries and declining fish resources [6], and has been stagnant to date. In addition, a substantial amount of FM has been incorporated in feeds for terrestrial livestock [7]. Consequently, the limited availability and increasing competition of FM indicate that aquaculture will be constrained by a significant bottleneck in the near future, as long as the fish feed industry depends on the availability of FM. 

Several attempts have been made to evaluate various animal protein sources [8,9,10] and plant protein source [8,11,12,13] as the substitutes for FM in the red sea bream diets, with substantial achievements. However, the relatively low protein level, imbalanced amino acid (AA) composition, the presence of anti-nutritional factors, and poor digestibility of plant protein sources have limited their widespread use in diets, especially for carnivorous fish species [14,15]. Thus, it is necessary for feed nutritionists to search for an animal-origin replacer, which is free of these issues, for FM in fish feeds.

Fishery by-products are increasingly considered the practical replacer for FM in aquafeeds [16,17]. In fish processing plants, over 60% of fishery by-products, including heads, skin, trimmings, fins, frames, viscera, and roes are generated as waste, and only 40% of fish products are produced for human consumption [18]. The disposal of large amounts of these by-products can cause highly polluting organic matter, which leads to environmental and economic issues [19,20]. However, fishery by-products represent excellent sources of high-quality protein and lipids, along with being rich in micronutrients, such as vitamins (A, B2, B3, and D) and minerals (iron, zinc, selenium, and iodine) [21]. Tuna by-product is a type of fishery by-product generated from the canning process of the primary market species of tuna, such as skipjack tuna (*Katsuwonus pelamis*) and yellowfin tuna (*Thunnus albacares*) [22,23]. In recent years, the tuna cannery industry has been increasingly exploring the utilization of by-products generated during tuna processing to innovate new products and enhance profit margins [22]. As a result, various feed ingredients derived from tuna by-products, including tuna by-product meal (TBM), tuna silage, and tuna protein hydrolysate, have been developed [22].

Previous studies have reported the potential for substituting FM with TBM in the diets of olive flounder (*Paralichthys olivaceus*) [23], spotted rose snapper (*Lutjanus guttatus*) [24], and rockfish (*Sebastes schlegeli*) [25]. Additionally, Uyan et al. [26] found that tuna muscle by-product powder (TMP) (obtained after the de-boning process of TBM) was an appropriate protein source to replace FM up to 50% in the 58.5% FM-basal diet without adverse impacts on growth of red sea bream. However, diets used in Uyan et al. [26]’s study contained relatively low protein contents (45−46%), which were lower than the dietary protein requirement (52%) of red sea bream [27], and the use of TMP in fish feeds is very restricted because of its limited supply and high cost [26,28]. Nevertheless, TBM produced from tuna canning process could be commercially available in Korea, with production of over 30,000 metric tons by Woojin Feed Ind. Co. Ltd. (Incheon Metropolitan City, Republic of Korea) in 2020 [23], although statistical data on the annual production of TBM was unavailable in Korea to date. Despite the commercial importance of TBM in fish feeds, no study on evaluation of the potential substitution of FM with TBM in the diet of red sea bream has been reported. 

This study, thus, aimed to evaluate effect of dietary FM replacement with TBM on the growth and feed availability, biochemical composition, and blood chemistry of red sea bream. Additionally, the economic effect of dietary substitution of FM with TBM was also investigated.

## 2. Materials and Methods

### 2.1. Experimental Fish and Conditions

Similar sizes of juvenile red sea bream were obtained from a commercial fish farm (Tongyeong-si, Chungcheongnam-do, Republic of Korea) and acclimated in a 5-ton round shaped tank for 2 weeks. During this period, they were provided with a commercial extruded pellet (50% crude protein and 13% crude lipid) (Suhyup Feed, Uiryeong-gun, Gyeongsangnam-do, Republic of Korea). After acclimation, 900 juveniles averaging 8.6 g were allocated into 18, 300-L flow-through circular tanks (50 fish/tank) in triplicate for the 8-week feeding trial. The tanks were filled with a 1:1 mixture of sand-filtered seawater and underground seawater. Water quality was monitored daily throughout the feeding experiment using a digital multimeter (AZ-8603, AZ Instrument, Taichung, Taiwan). The water temperature, dissolved oxygen, salinity, and pH were recorded at 20.6 ± 1.54 °C (mean ± SD), 7.7 ± 0.27 mg/L, 30.5 ± 0.40 g/L, and 7.5 ± 0.07, respectively. Fish were meticulously hand-fed to visual satiation twice a day (08:00 and 17:00). The experimental conditions were maintained under the natural photoperiod. To maintain adequate water quality, the bottom of each tank underwent daily siphon-cleaning, and deceased fish were promptly removed upon detection.

### 2.2. Experimental Diets

The feed formulations of the experimental feeds are shown in Table 1. The control (Con) diet contained 55% FM (anchovy meal) and 17% soybean meal as the protein sources. Additionally, 17.5% wheat flour and 4% each of fish and soybean oils were contained as the carbohydrate and lipid sources, respectively, in the Con diet. In the Con diet, TBM was replaced for 20, 40, 60, 80, and 100% of FM, designated as the TBM20, TBM40, TBM60, TBM80, and TBM100 diets, respectively. All experimental diets were isonitrogenous at 51.5% and isolipidic at 14.5%. The experimental diets were formulated to fulfill protein and lipid requirements for red sea bream [27]. All feed ingredients were finely pulverized, thoroughly mixed, and pelleted using a laboratory pellet extruder with a 3:1 water ratio. The experimental feeds were dried at 40 °C for a couple of days, and stored at −20 °C until use.

### 2.3. Measurement of Biological Indices of Fish

On the completion of the feeding trial, all live red sea bream in each tank were starved for 24 h and then anesthetized with tricaine methanesulfonate (MS-222) at a concentration of 100 ppm. The total number of fish in each tank was counted and the collective weight was measured. In each tank, ten anesthetized fish were randomly selected to calculate biological indices, including condition factor (CF), viscerosomatic index (VSI), and hepatosomatic index (HSI). Growth performance, feed utilization, and biological indices were calculated as follows [28]: specific growth rate (SGR, %/day) = (Ln final weight of fish − Ln initial weight of fish) × 100/days of feeding trial (56 days); feed efficiency (FE) = (Total final weight (g) − total initial weight (g) + total weight of dead fish (g)]/total feed consumption (g)); protein efficiency ratio (PER) = weight gain of fish (g/fish)/total protein consumption of fish (g/fish); protein retention (PR, %) = protein gain of fish (g/fish) × 100/total protein consumption of fish (g/fish), CF (g/cm^3^) = body weight of fish (g) × 100/total length of fish (cm)^3^; VSI (%) = viscera weight of fish (g) × 100/body weight of fish (g); and HSI (%) = liver weight of fish (g) × 100/body weight of fish (g).

### 2.4. Blood Chemistry of Red Sea Bream

Blood samples were collected from five anesthetized fish from each tank using heparinized syringes after the measurement of individual weight to determine the biological indices. After centrifugation (2720× *g*) at 4 °C for 10 min, plasma samples were collected and stored at −70 °C in separate aliquots. These samples were later analyzed for aspartate aminotransferase (AST), alanine aminotransferase (ALT), alkaline phosphatase (ALP), total bilirubin (T-BIL), total cholesterol (T-CHO), triglyceride (TG), total protein (TP), and albumin (ALB) levels using an automatic chemistry system (Fuji Dri-Chem NX500i, Fujifilm, Tokyo, Japan).

Blood samples were collected from five anesthetized fish from each tank using syringes after measurement of individual weight to determine the biological indices. After centrifugation (2720× *g*) at °C for 10 min, serum samples were collected and stored at −7 °C in separate aliquots. Superoxide dismutase (SOD) was determined in terms of the percentage reaction inhibition rate of enzyme with water-soluble tetrazolium dye (WST-1) as the substrate and xanthine oxidase using a SOD assay kit (Sigma, 19160, St. Louis, MO, USA), following the standard protocol. After incubation at 3 °C for 20 min, the absorbance of each endpoint assay was measured at 450 nm, which is the absorbance wavelength for the colored product of the WST-1 reaction with superoxide. The inhibition percentage was normalized by mg protein and expressed as SOD units.

Furthermore, turbidimetric assay for lysozyme was performed as described by Lange et al. [30]. In short, a 100 µL of test serum was introduced into a 1.9 mL suspension of *Micrococcus lysodeikticus* (0.2 mg/mL; Sigma, St. Louis, MO, USA) in 0.05 M sodium phosphate buffer (pH 6.2). The reactions were conducted at 25 °C, and absorbance at 530 nm was assessed using a spectrophotometer between 0 and 60 min. The lysozyme activity unit was calculated as the amount of enzyme required to produce a 0.001/min reduction in absorbance.

### 2.5. Analysis of Biochemical Composition of the Experimental Feeds and Fish

The proximate composition of the experimental feeds and the whole-body fish were analyzed according to standard protocol [31]. Moisture content was measured by oven drying at 105 °C (6 h for dry samples and 24 h for wet samples). Crude protein content was analyzed using the Kjeldahl method (Kjeltec 2100 Distillation Unit, Foss Tecator, Hoganas, Sweden), while crude lipid content was analyzed using an ether-extraction method (Soxtec TM 2043 Fat Extraction System, Foss Tecator, Sweden). Ash content was analyzed using a muffle furnace operated at 550 °C for 4 h.

Analysis of All AA (except tryptophan) in the FM, TBM, experimental feeds, and whole body of red sea bream were conducted using the ninhydrin postcolumn reaction method through ion-exchange chromatography with an AA analyzer (L-8800 Auto-analyzer, Hitachi, Tokyo, Japan). For each sample, 0.2 g was placed in a decomposition tube, 10 mL of 6 N HCl was added, and then the mixture was hydrolyzed at 110 °C for 24 h with nitrogen gas injection. The filtrate was concentrated using a reduced-pressure concentrator, adjusted to a volume of 50 mL with 0.2 M sodium citrate buffer, and filtered through a 0.20 μm cellulose acetate syringe filter before analysis. Tryptophan content was measured separately using high-performance liquid chromatography (S1125 HPLC pump system, Sykam GmbH, Eresing, Germany).

Fatty acid (FA) in the experimental feeds and whole body of red sea bream were extracted using a mixture of chloroform and methanol (2:1), as described by Folch et al. [32]. FA methyl esters were synthesized through transesterification with 14% BF3-MeOH (Sigma, St. Louis, MO, USA) and subjected to analysis using a gas chromatograph (Trace GC, Thermo, Waltham, MA, USA) equipped with a flame ionization detector. The separation was carried out on an SPTM-2560 capillary column (100 m × 0.25 mm I.d. film thickness 0.20 µm; Supelco, Bellefonte, PA, USA).

### 2.6. Analysis of Economic Measurements of the Study

The economic evaluation of this study was conducted using USD as the currency type. The economic conversion ratio (ECR) and economic profit index (EPI) were calculated according to Bicudo et al. [33] and Montenegro et al. [34]: ECR (USD/kg) = feed consumption of fish (kg/fish)/weight gain of fish (kg/fish) × diet price (USD/kg) and EPI (USD/fish) = [final weight of fish (kg/fish) × selling price of fish (USD/kg)] − [feed consumption of fish (kg/fish) × diet price (USD/kg)]. The prices of feed ingredients and fish were calculated using the exchange rate USD 1 = KRW 1232 (Korean currency). The price of red sea bream was estimated at 20.29 USD/kg. The price of the experimental diets was computed by multiplying the proportional contributions of each feed ingredient by their respective cost per kg and summing the resulting values for all ingredients in the experimental diets. The price (USD/kg) of each ingredient was as follows: FM = 2.16; TBM = 1.30; fermented soybean meal = 0.70; wheat flour = 0.55; fish oil = 2.76; soybean oil = 1.79; vitamin premix = 8.28; mineral premix = 6.66; choline = 1.30.

### 2.7. Statistical Analysis

All statistical analyses were carried out using SPSS version 24.0 (SPSS Inc., Chicago, IL, USA). Data were evaluated for assumptions including normality and homogeneity of variance, using the Shapiro–Wilk and Levene tests, respectively, and violation was not detected (*p* > 0.05). One-way analysis of variance (ANOVA) and Duncan’s multiple range test [35] were used to compare the means of dietary treatments. Percentage data were arcsine-transformed before statistical analysis. If statistical significance was detected (*p* < 0.05), the data were subjected to orthogonal polynomial contrast and regression analysis to determine the most suitable model (linear, quadratic, and cubic).

## 3. Results

### 3.1. Amino Acid and Fatty Acid Profiles of the Experimental Feeds 

FM contained relatively high content of all essential AA (EAA) and non-essential AA (NEAA), except for glycine compared over TBM (Table 2). As dietary FM replacement levels with TBM increased, the content of arginine increased, while all EAA except for phenylalanine and threonine tended to decrease.

TBM contained relatively high content total content of n-3 highly unsaturated FA (∑n-3 HUFA), including docosahexaenoic acid (DHA, C22:6n-3), but low content of total content of monounsaturated FA (∑MUFA) and eicosapentaenoic acid (EPA, C20:5n-3) over FM (Table 3). Additionally, with an increase in the substitution levels of FM by TBM, the content of ∑n-3 HUFA in the experimental feeds tended to increase, whereas the content of total content of saturated FA (∑SFA) and ∑MUFA tended to decrease.

### 3.2. Performance of Fish

The survival of fish was not statistically (*p* > 0.9) altered by dietary FM substitution by TBM (Table 4). Red sea bream fed the Con, TBM20, and TBM40 diets showed statistically (*p* < 0.0001) superior weight gain to fish fed all other diets. Weight gain of fish fed the TBM60 and TBM80 diets was also statistically (*p* < 0.05) higher than that of fish fed the TBM100 diet. The SGR of red sea bream fed the Con diet was statistically (*p* < 0.0001) superior to fish fed the TBM60, TBM80, and TBM100 diets, but comparable to fish fed the TBM20 and TBM40 diets. Additionally, polynomial orthogonal contrast showed significant linear (*p* = 0.0206 and *p* = 0.0236, respectively) and quadratic (*p* = 0.0001 for both) models between dietary replacement levels of TBM for FM versus weight gain and SGR (Table 5). In regression analysis, quadratic relationships were suggested as the most suitable relationships between dietary substitution levels of TBM for FM versus weight gain (Y = −0.000595X^2^ + 0.009619X + 32.9571, *p* < 0.0001, R^2^ = 0.8335, Y_max_ = X value of 8.1%) and SGR (Y = −0.000027X^2^ + 0.00043X + 2.8092, *p* < 0.0001, R^2^ = 0.8263, Y_max_ = X value of 8.0%).

### 3.3. Feed Consumption, Feed Utilization, and Biological Indices of Red Sea Bream

The feed consumption (g/fish) of red sea bream fed the Con diet was statistically (*p* < 0.03) higher than that of red sea bream fed the TBM60, TBM80, and TBM100 diets, but not statistically (*p* > 0.05) different from that of red sea bream fed the TBM20 and TBM40 diets (Table 6). Polynomial orthogonal contrast showed significant liner (*p* = 0.0009) model between dietary replacement levels of TBM for FM and feed consumption. In regression analysis, a linear relationship was suggested as the most suitable relationship between dietary FM substitution with TBM and feed consumption (Y = −0.030333X + 36.0322, *p* < 0.0001, R^2^ = 0.5833).

The FE of red sea bream fed the Con, TBM20, TBM40, TBM60, and TBM80 diets was statistically (*p* < 0.04) higher than that of red sea bream fed the TBM100 diet. Polynomial orthogonal contrast showed a significant quadratic (*p* = 0.0040) model between dietary replacement levels of TBM for FM and FE. In regression analysis, a quadratic relationship was suggested as the most suitable relationship between dietary FM substitution with TBM and FE (Y = −0.00001308X^2^ + 0.000559X + 0.9685, *p* < 0.003, R^2^ = 0.5473, Y_max_ = X value of 21.4%). The PER of red sea bream fed the TBM20 and TBM40 diets was statistically (*p* < 0.006) higher than that of red sea beam fed the TBM80 and TBM100 diets, but not statistically (*p* > 0.05) different from that of red sea beam fed the Con and TBM60 diets. The PR of red sea bream fed the TBM20, TBM40, and TBM60 diets was statistically (*p* < 0.002) higher than that of red sea bream fed the TBM80 and TBM100 diets, but not statistically (*p* > 0.05) different from that of red sea bream fed the Con diet. Polynomial orthogonal contrast showed significant linear (*p* = 0.0051 and *p* = 0.0022, respectively) and quadratic (*p* = 0.0014 and *p* = 0.0001, respectively) models between dietary substitution levels of TBM for FM and PER and PR. In regression analysis, quadratic relationships were suggested as the most suitable relationships between dietary FM substitution with TBM and PER (Y = −0.00003869X^2^ + 0.002507X + 1.7743, *p* < 0.0001, R^2^ = 0.6815, Y_max_ = X value of 32.1%) and PR (Y = −0.000935X^2^ + 0.068738X + 28.4619, *p* < 0.0001, R^2^ = 0.7798, Y_max_ = X value of 36.8%).

Biological indices of fish, including CF (1.75–1.87 g/cm^3^), VSI (8.69–9.06%), and HSI (2.32–2.75%) were not remarkably (*p* > 0.6, *p* > 0.8, and *p* > 0.06, respectively) changed by dietary treatment.

### 3.4. Blood Chemistry Parameters of Fish

Plasma levels of AST, ALT, ALP, T-BIL, T-CHO, TG, TP, and ALB were in the ranges of 49.4–53.3 U/L, 7.3–9.1 U/L, 167.0–170.0 U/L, 0.9–1.2 mg/dL, 249.6–252.2 mg/dL, 389.6–408.7 mg/dL, 4.6–5.0 g/dL, and 0.9–1.2 g/dL, respectively (Table 7). The experimental diets had no remarkable (*p* > 0.05 for all) effects on these parameters. 

Serum lysozyme activity and SOD of red sea bream ranged 67.0–67.9 U/mL and 34.9–43.0%, respectively. None of these parameters was statistically (*p* > 0.8 for both) altered by dietary TBM substitution for FM.

### 3.5. Biochemical Composition of the Whole Body of Red Sea Bream 

The content of moisture, crude protein, crude lipid, and ash were in the ranges of 68.4–69.5%, 16.1–16.5%, 8.4–9.0%, and 4.5–4.8%, respectively (Table 8). None of these parameters was statistically (*p* > 0. 8, *p* > 0.7, *p* > 0.7, and *p* > 0.9, respectively) altered by dietary TBM substitution for FM.

The whole-body AA (Table 9) and FA (Table 10) profiles of red sea bream were remarkably (*p* > 0.05 for all) unaffected by dietary TBM substitution for FM.

### 3.6. Economic Analysis of the Study

Diet price and ECR were the highest in the Con diet (Table 11). Price and ECR of the diets decreased with elevated dietary FM replacement with TBM. EPI of the TBM40 diet was statistically (*p* < 0.0001) higher than that of the TBM60, TBM80, and TBM100 diets, but not statistically (*p* > 0.05) different from that of the Con and TBM20 diets. Polynomial orthogonal contrast showed significant linear (*p* = 0.0122 and *p* = 0.0085, respectively) and quadratic (*p* = 0.0001 for both) models between dietary replacement levels of TBM for FM versus ECR and EPI. In regression analysis, quadratic relationships were suggested as the most suitable relationships between dietary FM replacement with TBM and ECR (Y = 0.000026X^2^ − 0.005403X + 1.5146, *p* < 0.0001, R^2^ = 0.9014) and EPI (Y = −0.000014X^2^ + 0.00057X + 0.7783, *p* < 0.0001, R^2^ = 0.7778).

## 4. Discussion

The utilization of fishery by-products including TBM as a protein source in commercial fish feeds can be the economical and practical solutions for mitigating environmental concerns and reducing feed cost [21,22,23]. There were no discernible changes in weight gain and SGR of red sea bream fed the Con, TBM20, and TBM40 diets in this study, which implied that FM replacement up to 40% by TBM in diets led to no undesirable impact on growth of fish. This finding is in accordance with previous studies, in which the substitution of FM with TBM up to 50% and 75% in the feeds of olive flounder [23] and spotted rose snapper [24], and rockfish [25], respectively, did not compromise the growth performance. Likewise, Uyan et al. [26] demonstrated that substituting FM up to 50% with TMP did not adversely change the growth of red sea bream. Previous studies also reported that dietary substitution of tuna liver meal and TBM for FM up to 30 and 75%, respectively, is feasible without negatively affecting growth of Nile tilapia (*Oreochromis niloticus*) and abalone (*Haliotis discus*), respectively [40,41]. Furthermore, a blend of tuna viscera and corn meal (65:35) was also used as an alternative for FM in the feed for white shrimp (*Litopenaeus vannamei*), and FM up to 40% could be substitutable without any detrimental effect on growth [42].

All EAA, except for arginine, phenylalanine, and threonine, decreased with elevated replacement of FM by tuna by-product meal TBM. The arginine, lysine, and valine requirements for the growth of red sea bream have been reported to be 2.37% [36], 1.79% [37], and 0.90% [38] of diets, respectively. Arginine (2.58–2.72%), lysine (3.46–3.68%), and valine (2.12–2.30%) levels in all experimental diets were met for their requirements. Unfortunately, the requirements for most of the EAA remain unknown, making it difficult to clearly explain the effects of the deficiency of each EAA on the growth performance of red sea bream. Although a few EAA (arginine, lysine, and valine) requirements for red sea bream were known, decreased content of ∑EAA in the experimental diets, especially in the TBM60, TBM80, and TBM100 diets, could appear to partially affect the growth of red sea bream negatively.

Marine fish species typically require dietary n-3 HUFA, such as EPA and DHA, for desirable growth and survival [42]. The dietary requirements of EPA and DHA for juvenile red sea bream were estimated to be 1% (6.85% of total FA) and 0.5% (3.42% of total FA), respectively, when either DHA or EPA was not present, respectively [43]. However, when EPA and DHA at a ratio of 1:1 was included in feeds, their requirements could be reduced by 0.25% (1.71% of total fatty acids) in diets for each. Therefore, the experimental diets appeared to fulfill the dietary requirements of both EPA and DHA for red sea bream in this study.

A linear decrease in feed consumption was observed in red sea bream with dietary elevated levels of FM substitution with TBM in regression analysis, and red sea bream fed the TBM60, TBM80, and TBM100 diets showed statistically lower feed consumption than that of fish fed the Con diet in this study, implying that lower feed consumption of fish fed the formers led to poorer growth compared to fish fed the latter. Likewise, low feed consumption, attributed to poor palatability has been reported when high amount of FM was replaced with various animal protein sources in fish feeds [44,45,46]. Increased ash content from 9.8% to 13.6% in the experimental diets with elevated FM substitution with TBM could be a reason why red sea bream produced poorer growth and feed consumption in the higher FM-substituted diets in this study. The administration of diets containing high ash content led to adverse effect on fish performances, such as poor growth, high mortality, cataracts, and skeletal abnormalities [47,48,49]. A significant reduction in growth rate resulted from lower feed consumption was also observed in chinook salmon (*Oncorhynchus tshawytscha*) fed the feeds containing higher levels of calcium and phosphorus (dietary ash content of 19.3–19.4%) compared to fish fed a diet without calcium and phosphorus supplementation (dietary ash content of 3.5–3.6%) in the 105-day feeding trial [50].

The Y_max_ values to induce the greatest FE, PER, and PR of red sea bream were estimated to be 21.4, 32.1, and 36.8% of FM replacement by TBM in diets, whereas the Y_max_ values to induce the greatest weight gain and SGR were estimated to be 8.1 and 8.0% of FM replacement by TBM in this study, respectively. Results of the multiple comparison in growth performance (weight gain and SGR) of red sea bream seemed to be more similar to those of the Y_max_ values to achieve the greatest FE, PER, and PR, rather than those of the Y_max_ values to achieve weight gain and SGR in regression analysis in this study. 

CF has been commonly used as an indicator of the condition, fatness, and well-being of fish, and the heavier fish of a given length is generally considered to be the better condition [51]. VSI is an indicator of how lipids are being utilized and is positively correlated with dietary lipid levels [52]. HSI is a common method for indirectly measuring glycogen and carbohydrate levels accumulated in liver to evaluate the nutritional condition of fish [52,53]. In this study, dietary substitution of FM with TBM did not statistically alter CF, VSI, and HSI of red sea bream, implying that the health status of fish was not affected by dietary FM replacement with TBM. This was consistent with a study [23], in which dietary FM substitution with TBM led to no remarkable changes in the CF, VSI, and HSI of olive flounder. In addition, dietary FM replacement with fermented TBM led to no remarkable changes in the HSI and CF of olive flounder [54].

Plasma parameters are the critical indicators of fish’s health and physiological stress response [55,56]. No discernible differences in plasma parameters of red sea bream in this study implied that dietary FM substitution with TBM did not lead to any adverse effect on fish health. Likewise, Uyan et al. [26] reported that plasma parameters of red sea bream were not altered by dietary FM substitution with TMP. Similarly, dietary FM substitution with TBM did not bring about significant differences on plasma parameters in spotted rose snapper [24] and olive flounder [23]. However, in contrast to this study, Oncul et al. [54] emphasized that dietary FM replacement with fermented TBM significantly altered plasma AST and T-CHO of olive flounder.

Innate immunity constitutes a fundamental defense system in fish and is commonly used to evaluate the effects of dietary treatments on fish health and immune function [57,58]. Lysozyme plays a crucial role in protecting against infectious diseases by breaking down glycosidic bonds present in the peptidoglycan of cell walls, regardless of whether they are gram-positive or gram-negative [58,59]. SOD serves as a crucial antioxidant enzyme, protecting cells against oxidative damage caused by reactive oxygen species [60,61]. Dietary substitution of FM with TBM led to no discernible changes in lysozyme activity and SOD of fish in this study, implying that dietary FM replacement with TBM had no negative impact on serum lysozyme activity and SOD of red sea bream. Similarly, previous studies have shown that lysozyme activity and SOD in olive flounder were not altered by dietary FM substitution, regardless of whether unfermented or fermented TBM was used [23,54]. 

No significant differences in the proximate composition, AA, and FA profiles of red sea bream in this study indicated that dietary FM replacement with TBM did not cause any negative impact on the biochemical composition of fish. These findings were consistent with the findings of previous studies, in which dietary FM substitution with various animal protein sources did not cause any difference in the chemical composition [10,62,63,64] and AA profiles [25,65,66] of fish. The AA profiles of body proteins seem to be the same regardless of diets because body proteins are synthesized based on the genetic coding from DNA [67]. Unlike this study, however, the FA profiles of fish were changed by FM replacement with animal protein sources in fish feeds [62,68].

The price of the experimental diets and ECR decreased with elevated FM substitution with TBM in this study. However, the greatest EPI was achieved in the TBM40 diet. EPI is a crucial parameter to assess economic profitability in considering growth performance, feed consumption, feed cost, and fish selling prices [33,69]. Remarkably lower EPI in the TBM60, TBM80, and TBM100 diets compared to the TBM40 diet might be attributed to poorer growth of fish. Likewise, previous studies have proven that the greatest EPI can be achieved by FM replacement with cost-effective protein sources at appropriate level in fish feeds [33,69,70,71]. Therefore, 40% FM substitution with TBM in the red sea bream feed is anticipated to yield the greatest economic return for fish farmers. The feasibility of 40% FM substitution with TBM in the commercial feed of red sea bream needs to be tested in a long-term feeding trial. 

## 5. Conclusions

FM replacement up to 40% could be made with TBM in the 55% FM-based diet without adverse effects on the growth and feed availability of red sea bream. Furthermore, the greatest EPI was achieved in the TBM40 diet. 

## Figures and Tables

**Table 1:** Ingredients and chemical composition of the experimental diets (%, dry matter basis).

	Experimental Diets					
	Con	TBM20	TBM40	TBM60	TBM80	TBM100
Ingredients (%)						
Anchovy meal ^1^	55.0	44.0	33.0	22.0	11.0	0.0
Tuna by-product meal ^2^	0.0	13.2	26.4	39.5	52.7	65.8
Fermented soybean meal	17.0	17.0	17.0	17.0	17.0	17.0
Wheat flour	17.5	15.2	12.9	10.8	8.5	6.3
Fish oil	4.0	4.1	4.2	4.2	4.3	4.4
Soybean oil	4.0	4.0	4.0	4.0	4.0	4.0
Mineral mix ^3^	1.0	1.0	1.0	1.0	1.0	1.0
Vitamin mix ^4^	1.0	1.0	1.0	1.0	1.0	1.0
Choline	0.5	0.5	0.5	0.5	0.5	0.5
Nutrients (%)						
Dry matter	95.7	94.8	95.8	95.9	96.1	95.9
Crude protein	51.3	51.7	51.5	51.3	51.7	51.7
Crude lipid	14.6	14.5	14.7	14.8	14.4	14.4
Ash	9.8	10.5	11.3	12.4	13.0	13.6
Estimated energy (kcal/g) ^5^	4.3	4.3	4.3	4.2	4.2	4.2

^1^ Anchovy meal (crude protein: 73.4%, crude lipid: 10.7%, ash: 14.0%) was imported from Chile [USD 1.84/kg, USD 1 = 1302 KRW (Korean currency)]. ^2^ Tuna by-product meal (TBM) (crude protein: 64.0%, crude lipid: 8.9%, ash: 18.2%) was purchased from Woojin Feed Ind. Co. Ltd. (Incheon Metropolitan City, Korea) (USD 1.30/kg). ^3^ Mineral premix (g/kg mix): MgSO_4_·7H_2_O, 80.0; NaH_2_PO_4_·2H_2_O, 370.0; KCl, 130.0; ferric citrate, 40.0; ZnSO_4_·7H_2_O, 20.0; Ca-lactate, 356.5; CuCl, 0.2; AlCl_3_·6H_2_O, 0.15; KI, 0.15; Na_2_Se_2_O_3_, 0.01; MnSO_4_·H_2_O, 2.0; CoCl_2_·6H_2_O, 1.0. ^4^ Vitamin premix (g/kg mix): L-ascorbic acid, 121.2; DL-α-tocopheryl acetate, 18.8; thiamin hydrochloride, 2.7; riboflavin, 9.1; pyridoxine hydrochloride, 1.8; niacin, 36.4; Ca-D-pantothenate, 12.7; myo-inositol, 181.8; D-biotin, 0.27; folic acid, 0.68; p-aminobenzoic acid, 18.2; menadione, 1.8; retinyl acetate, 0.73; cholecalciferol, 0.003; cyanocobalamin, 0.003. ^5^ Estimated energy was calculated based on 4 kcal/g for protein and carbohydrate, and 9 kcal/g for lipid [29].

**Table 2:** Amino acid profiles (% of the diet) of the experimental diets.

	Ingredients		Requirement	Experimental Diets					
	FM	TBM		Con	TBM20	TBM40	TBM60	TBM80	TBM100
Essential amino acids (EAA)									
Arginine	3.87	3.46	2.37 ^3^	2.58	2.60	2.62	2.65	2.68	2.72
Histidine	1.89	1.56		1.38	1.36	1.35	1.35	1.33	1.32
Isoleucine	2.89	2.10		1.70	1.65	1.63	1.63	1.62	1.57
Leucine	5.03	3.99		3.82	3.76	3.72	3.65	3.60	3.55
Lysine	5.36	4.06	1.79 ^4^	3.68	3.64	3.61	3.55	3.51	3.46
Phenylalanine	2.64	2.18		2.12	2.16	2.15	2.12	2.12	2.11
Threonine	2.95	2.59		2.30	2.27	2.27	2.30	2.27	2.31
Tryptophan	0.69	0.41		0.36	0.35	0.34	0.32	0.29	0.29
Valine	3.34	2.58	0.90 ^5^	2.30	2.25	2.23	2.19	2.16	2.12
∑EAA ^1^	28.66	22.93		20.24	20.04	19.92	19.76	19.58	19.45
Non-essential amino acids (NEAA)									
Alanine	4.16	3.93		2.76	2.79	2.84	2.88	2.92	2.96
Aspartic acid	6.07	5.00		4.95	4.95	4.93	4.93	4.95	4.92
Glutamic acid	8.34	7.08		6.34	6.32	6.30	6.28	6.25	6.24
Glycine	3.76	4.00		2.94	3.05	3.13	3.21	3.24	3.36
Proline	2.74	2.69		2.16	2.18	2.20	2.23	2.24	2.27
Serine	2.58	2.34		1.72	1.73	1.77	1.82	1.88	1.95
Tyrosine	1.82	1.41		1.39	1.36	1.35	1.34	1.32	1.28
∑NEAA ^2^	29.47	26.45		22.26	22.38	22.52	22.69	22.80	22.98

^1^ ∑EAA: Total content of essential amino acid. ^2^ ∑NEAA: Total content of non-essential amino acid. Arginine ^3^, lysine ^4^, and valine ^5^ requirements were obtained from Rahimnejad and Lee [36], Forster and Ogata [37], and Rahimnejad and Lee [38], respectively.

**Table 3:** Fatty acid profiles (% of total fatty acids) of the experimental diets.

	Ingredients		Requirement	Experimental Diets					
	FM	TBM		Con	TBM20	TBM40	TBM60	TBM80	TBM100
C14:0	5.09	4.25		2.49	2.37	2.35	2.27	2.25	2.22
C16:0	23.06	25.02		15.72	15.79	15.89	16.00	16.09	16.18
C18:0	8.05	6.59		4.77	4.66	4.60	4.52	4.40	4.25
C20:0	0.98	0.76		0.76	0.70	0.68	0.65	0.63	0.62
C22:0	0.30	0.34		0.34	0.32	0.31	0.34	0.35	0.37
C24:0	0.68	1.14		0.48	0.51	0.52	0.53	0.55	0.56
∑SFA ^1^	38.16	38.10		24.56	24.35	24.35	24.31	24.27	24.20
C14:1n-5	0.23	0.12		0.10	0.07	0.08	0.08	0.08	0.08
C15:1n-5	0.15	0.18		0.10	0.09	0.08	0.07	0.08	0.09
C16:1n-7	5.47	4.09		3.14	2.84	2.74	2.69	2.58	2.50
C17:1n-7	0.78	0.73		0.38	0.40	0.40	0.41	0.43	0.44
C18:1n-9	23.30	22.16		30.22	30.14	29.97	29.74	29.50	29.35
C20:1n-9	1.01	2.73		1.01	1.05	1.14	1.15	1.18	1.20
C22:1n-9	0.19	0.31		0.38	0.40	0.41	0.42	0.43	0.43
C24:1n-9	2.69	1.23		1.08	1.02	0.95	0.80	0.75	0.70
∑MUFA ^2^	33.82	31.55		36.41	36.01	35.77	35.36	35.03	34.79
C18:2n-6	1.89	2.50		23.10	23.15	23.19	23.33	23.40	23.44
C18:3n-3	0.70	0.82		3.74	3.74	3.75	3.77	3.80	3.82
C18:3n-6	0.30	0.44		0.39	0.59	0.60	0.61	0.61	0.62
C20:2n-6	0.07	0.04		0.07	0.03	0.02	0.03	0.03	0.04
C20:3n-3	0.17	0.00		0.02	0.01	0.02	0.02	0.01	0.01
C20:3n-6	0.08	0.00		0.02	0.02	0.03	0.03	0.02	0.03
C20:4n-6	2.44	3.35		0.73	0.91	0.95	1.00	1.12	1.19
C20:5n-3	7.06	5.22	6.85 ^4^	2.82	2.68	2.63	2.55	2.50	2.44
C22:2n-6	0.60	0.52		0.39	0.36	0.36	0.36	0.36	0.35
C22:6n-3	14.71	17.46	3.42 ^4^	4.87	5.05	5.14	5.59	6.28	6.45
∑n-3 HUFA ^3^	21.94	22.68		7.71	7.74	7.79	8.16	8.79	8.90
Unknown	0.08	0.09		2.88	3.10	3.19	3.04	2.57	2.62

^1^ ∑SFA: Total content of saturated fatty acid. ^2^ ∑MUFA: Total content of monounsaturated fatty acid. ^3^ ∑ n-3 HUFA: Total content of n-3 highly unsaturated fatty acid. EPA (C20:5n-3) ^4^ and DHA (C22:6n-3) ^4^ requirements were obtained from Takeuchi et al. [39].

**Table 4:** Survival (%), weight gain (g/fish), and specific growth rate (SGR, %/day) of red sea bream fed the experimental diets for 8 weeks.

Experimental Diets	Initial Weight (g/Fish)	Final Weight (g/Fish)	Survival (%)	Weight Gain (g/Fish)	SGR ^1^ (%/Day)
Con	8.6 ± 0.01	41.5 ± 0.74 ^a^	91.3 ± 1.33	32.9 ± 0.73 ^a^	2.81 ± 0.030 ^a^
TBM20	8.7 ± 0.07	41.6 ± 0.60 ^a^	92.7 ± 4.37	32.9 ± 0.57 ^a^	2.79 ± 0.023 ^ab^
TBM40	8.6 ± 0.02	41.3 ± 0.45 ^a^	94.0 ± 3.06	32.7 ± 0.47 ^a^	2.79 ± 0.024 ^ab^
TBM60	8.6 ± 0.03	39.6 ± 0.26 ^b^	93.3 ± 3.53	31.0 ± 0.28 ^b^	2.73 ± 0.016 ^bc^
TBM80	8.6 ± 0.00	38.7 ± 0.36 ^b^	92.7 ± 2.91	30.1 ± 0.36 ^b^	2.69 ± 0.016 ^c^
TBM100	8.6 ± 0.01	36.6 ± 0.74 ^c^	94.0 ± 2.00	28.0 ± 0.73 ^c^	2.58 ± 0.035 ^d^
*p*-value	*p* > 0.5	*p* < 0.0001	*p* > 0.9	*p* < 0.0001	*p* < 0.0001

Values (means of triplicate ± SE) in the same column sharing the same superscript letter are not significantly different (*p* > 0.05). ^1^ SGR (%/day) = (Ln final weight of fish − Ln initial weight of fish) × 100/days of feeding trial.

**Table 5:** Relationship between dietary substitution levels of tuna by-product for fish meal versus growth performance (weight gain and SGR), feed availability (feed consumption, FE, PER, and PR), and economic parameters (ECR and EPI).

	Polynomial Orthogonal Contrast			Regression Analysis			
	Linear	Quadratic	Cubic	Equation	*p*-Value	R^2^	Y_max_ (%)
Growth performance							
Weight gain	0.0206	0.0001	0.8866	Y = −0.000595X^2^ + 0.009619X + 32.9571	0.0001	0.8335	X = 8.1
SGR	0.0236	0.0001	0.6847	Y = −0.000027X^2^ + 0.00043X + 2.8092	0.0001	0.8263	X = 8.0
Feed availability							
Feed consumption	0.0009	0.7711	0.3597	Y = −0.030333X + 36.0322	0.0001	0.5833	
FE	0.0953	0.0040	0.5812	Y = −0.00001308X^2^ + 0.000559X + 0.9685	0.003	0.5473	X = 21.4
PER	0.0051	0.0014	0.4155	Y = −0.00003869X^2^ + 0.002507X + 1.7743	0.0001	0.6815	X = 32.1
PR	0.0022	0.0001	0.4562	Y = −0.000935X^2^ + 0.068738X + 28.4619	0.0001	0.7798	X = 36.8
Economic parameter							
ECR	0.0122	0.0001	0.1912	Y = 0.000026X^2^ − 0.005403X + 1.5146	0.0001	0.9014	
EPI	0.0085	0.0001	0.5640	Y = −0.000014X^2^ + 0.00057X + 0.7783	0.0001	0.7778	

**Table 6:** Feed consumption, feed efficiency (FE), protein efficiency ratio (PER), protein retention (PR), condition factor (CF), viscerosomatic index (VSI), and hepatosomatic index (HSI) of red sea bream fed the experimental diets for 8 weeks.

Experimental Diets	Feed Consumption (g/Fish)	FE ^1^	PER ^2^	PR ^3^	CF ^4^	VSI ^5^	HIS ^6^
Con	36.29 ± 0.782 ^a^	0.97 ± 0.004 ^a^	1.77 ± 0.022 ^ab^	28.49 ± 0.353 ^ab^	1.87 ± 0.020	8.79 ± 0.306	2.75 ± 0.212
TBM20	35.20 ± 0.701 ^ab^	0.97 ± 0.024 ^a^	1.81 ± 0.032 ^a^	29.39 ± 0.527 ^a^	1.82 ± 0.054	7.06 ± 0.106	2.64 ± 0.045
TBM40	34.66 ± 0.558 ^abc^	0.97 ± 0.002 ^a^	1.83 ± 0.039 ^a^	29.84 ± 0.628 ^a^	1.86 ± 0.136	8.86 ± 0.166	2.32 ± 0.075
TBM60	33.98 ± 0.032 ^bc^	0.95 ± 0.025 ^a^	1.78 ± 0.017 ^ab^	29.39 ± 0.273 ^a^	1.89 ± 0.033	9.91 ± 0.421	2.57 ± 0.066
TBM80	34.16 ± 0.723 ^bc^	0.94 ± 0.015 ^a^	1.71 ± 0.023 ^bc^	27.61 ± 0.371 ^b^	1.75 ± 0.077	8.69 ± 0.080	2.36 ± 0.003
TBM100	32.81 ± 0.302 ^c^	0.89 ± 0.022 ^b^	1.65 ± 0.031 ^c^	26.13 ± 0.492 ^c^	1.77 ± 0.017	9.01 ± 0.186	2.49 ± 0.031
*p*-value	*p* < 0.03	*p* < 0.04	*p* < 0.006	*p* < 0.002	*p* > 0.6	*p* > 0.8	*p* > 0.06

Values (means of triplicate ± SE) in the same column sharing the same superscript letter are not significantly different (*p* > 0.05). ^1^ Feed efficiency (FE) = [Total final weight (g) − total initial weight (g) + total weight of dead fish (g)]/total feed consumption (g). ^2^ Protein efficiency ratio (PER) = weight gain of fish (g/fish)/total protein consumption of fish (g/fish). ^3^ Protein retention (PR, %) = protein gain of fish (g/fish) × 100/total protein consumption of fish (g/fish). ^4^ Condition factor (CF, g/cm^3^) = body weight of fish (g) × 100/total length of fish (cm)^3^. ^5^ Viscerosomatic index (VSI, %) = viscera weight of fish (g) × 100/body weight of fish (g). ^6^ Hepatosomatic index (HSI, %) = liver weight of fish (g) × 100/body weight of fish (g).

**Table 7:** Blood chemistry parameters of red sea bream fed the experimental diets for 8 weeks.

	Experimental Diets						
	Con	TBM20	TBM40	TBM60	TBM80	TBM100	*p*-Value
Plasma parameters							
AST (U/L)	50.9 ± 0.97	53.3 ± 1.93	49.4 ± 0.97	53.1 ± 1.89	51.2 ± 0.73	49.4 ± 1.14	*p* > 0.8
ALT (U/L)	9.1 ± 0.63	8.6 ± 0.39	8.2 ± 0.36	8.8 ± 0.58	7.3 ± 0.25	8.2 ± 0.64	*p* > 0.8
ALP (U/L)	168.9 ± 1.60	170.0 ± 2.30	167.8 ± 2.28	169.3 ± 1.68	167.8 ± 2.03	167.0 ± 2.42	*p* > 0.9
T-BIL (mg/dL)	0.9 ± 0.05	1.2 ± 0.14	1.2 ± 0.16	0.9 ± 0.05	1.0 ± 0.05	1.0 ± 0.06	*p* > 0.6
T-CHO (mg/dL)	251.6 ± 3.19	249.6 ± 3.89	252.2 ± 3.74	249.6 ± 0.80	250.2 ± 1.06	251.6 ± 4.67	*p* > 0.9
TG (mg/dL)	408.7 ± 11.17	389.6 ± 10.92	399.0 ± 4.58	395.7 ± 9.13	405.7 ± 10.30	392.4 ± 10.35	*p* > 0.8
TP (g/dL)	4.7 ± 0.09	4.6 ± 0.09	4.8 ± 0.08	4.7 ± 0.12	4.8 ± 0.08	5.0 ± 0.09	*p* > 0.8
ALB (g/dL)	1.1 ± 0.08	1.2 ± 0.09	0.9 ± 0.02	1.0 ± 0.03	1.2 ± 0.07	1.0 ± 0.03	*p* > 0.6
Serum parameters							
Lysozyme activity (U/mL)	67.9 ± 0.36	67.3 ± 0.36	67.3 ± 0.28	67.2 ± 0.22	67.0 ± 0.45	67.5 ± 0.36	*p* > 0.8
SOD (%)	34.9 ± 1.70	37.5 ± 5.46	38.0 ± 6.86	43.0 ± 7.87	35.0 ± 5.39	41.4 ± 4.77	*p* > 0.8

**Table 8:** Whole body proximate composition (% of wet weight) of red sea bream fed the experimental diets for 8 weeks.

Experimental Diets	Moisture	Crude Protein	Crude Lipid	Ash
Con	69.4 ± 0.98	16.1 ± 0.38	8.8 ± 0.39	4.6 ± 0.24
TBM20	69.3 ± 0.53	16.3 ± 0.26	9.0 ± 0.09	4.8 ± 0.22
TBM40	68.8 ± 0.49	16.3 ± 0.26	8.6 ± 0.19	4.5 ± 0.46
TBM60	68.4 ± 0.59	16.5 ± 0.40	8.9 ± 0.45	4.7 ± 0.10
TBM80	68.5 ± 1.03	16.2 ± 0.07	8.4 ± 0.44	4.5 ± 0.05
TBM100	69.5 ± 0.99	16.0 ± 0.13	8.6 ± 0.11	4.8 ± 0.13
*p*-value	*p* > 0.8	*p* > 0.7	*p* > 0.7	*p* > 0.9

**Table 9:** Amino acid profiles (% of wet weight) of red sea bream fed the experimental diets for 8 weeks.

	Experimental Diets						
	Con	TBM20	TBM40	TBM60	TBM80	TBM100	*p*-Value
Essential amino acids							
Arginine	1.00 ± 0.032	1.00 ± 0.020	0.96 ± 0.026	0.92 ± 0.020	0.95 ± 0.029	0.93 ± 0.032	*p* > 0.2
Histidine	0.34 ± 0.026	0.35 ± 0.015	0.37 ± 0.015	0.36 ± 0.032	0.36 ± 0.020	0.37 ± 0.017	*p* > 0.8
Isoleucine	0.52 ± 0.017	0.53 ± 0.015	0.53 ± 0.020	0.55 ± 0.026	0.52 ± 0.020	0.56 ± 0.017	*p* > 0.6
Leucine	1.12 ± 0.020	1.15 ± 0.029	1.13 ± 0.020	1.15 ± 0.026	1.11 ± 0.015	1.12 ± 0.015	*p* > 0.7
Lysine	1.26 ± 0.020	1.28 ± 0.023	1.27 ± 0.026	1.27 ± 0.015	1.24 ± 0.023	1.24 ± 0.026	*p* > 0.6
Phenylalanine	0.59 ± 0.026	0.60 ± 0.029	0.61 ± 0.015	0.61 ± 0.026	0.60 ± 0.017	0.61 ± 0.023	*p* > 0.9
Threonine	0.72 ± 0.020	0.74 ± 0.023	0.73 ± 0.020	0.73 ± 0.017	0.71 ± 0.032	0.73 ± 0.015	*p* > 0.9
Tryptophan	0.10 ± 0.017	0.11 ± 0.009	0.11 ± 0.015	0.09 ± 0.015	0.10 ± 0.015	0.10 ± 0.017	*p* > 0.9
Valine	0.67 ± 0.020	0.63 ± 0.015	0.63 ± 0.020	0.64 ± 0.015	0.62 ± 0.020	0.61 ± 0.012	*p* > 0.3
Non-essential amino acids							
Alanine	1.13 ± 0.023	1.07 ± 0.020	1.09 ± 0.023	1.05 ± 0.020	1.06 ± 0.023	1.05 ± 0.029	*p* > 0.2
Aspartic acid	1.49 ± 0.020	1.53 ± 0.026	1.50 ± 0.015	1.51 ± 0.023	1.47 ± 0.012	1.50 ± 0.017	*p* > 0.4
Glutamic acid	2.11 ± 0.017	2.09 ± 0.026	2.11 ± 0.020	2.13 ± 0.020	2.09 ± 0.023	2.11 ± 0.020	*p* > 0.7
Glycine	1.18 ± 0.020	1.19 ± 0.012	1.22 ± 0.020	1.23 ± 0.015	1.23 ± 0.017	1.24 ± 0.012	*p* > 0.1
Proline	0.72 ± 0.015	0.71 ± 0.015	0.70 ± 0.017	0.69 ± 0.020	0.70 ± 0.015	0.68 ± 0.020	*p* > 0.7
Serine	0.72 ± 0.017	0.75 ± 0.017	0.73 ± 0.017	0.72 ± 0.012	0.71 ± 0.017	0.73 ± 0.017	*p* > 0.6
Tyrosine	0.44 ± 0.020	0.47 ± 0.012	0.45 ± 0.015	0.45 ± 0.015	0.44 ± 0.012	0.43 ± 0.020	*p* > 0.6

**Table 10:** Fatty acid profiles (% of total fatty acids) of red sea bream fed the experimental diets for 8 weeks.

	Experimental Diets						
	Con	TBM20	TBM40	TBM60	TBM80	TBM100	*p*-Value
C14:0	2.66 ± 0.029	2.41 ± 0.115	2.03 ± 0.009	1.57 ± 0.165	1.99 ± 0.016	2.10 ± 0.343	*p* > 0.4
C16:0	15.59 ± 0.107	16.37 ± 0.360	15.75 ± 0.215	15.44 ± 0.412	15.90 ± 0.288	15.69 ± 0.108	*p* > 0.3
C18:0	4.58 ± 0.049	4.89 ± 0.153	4.84 ± 0.120	4.53 ± 0.191	4.60 ± 0.129	4.73 ± 0.198	*p* > 0.5
C20:0	1.07 ± 0.064	0.93 ± 0.067	0.93 ± 0.064	0.93 ± 0.035	0.82 ± 0.044	0.89 ± 0.050	*p* > 0.1
C22:0	0.80 ± 0.018	0.94 ± 0.035	1.05 ± 0.030	0.98 ± 0.133	1.03 ± 0.078	0.99 ± 0.043	*p* > 0.2
∑SFA ^1^	24.71 ± 0.266	25.53 ± 0.158	24.83 ± 0.464	24.35 ± 0.375	24.71 ± 0.354	24.74 ± 0.378	*p* > 0.3
C14:1n-5	0.10 ± 0.009	0.09 ± 0.018	0.08 ± 0.015	0.06 ± 0.015	0.06 ± 0.013	0.07 ± 0.015	*p* > 0.3
C15:1n-5	0.07 ± 0.009	0.06 ± 0.008	0.05 ± 0.015	0.04 ± 0.015	0.06 ± 0.018	0.05 ± 0.008	*p* > 0.7
C16:1n-7	4.52 ± 0.058	4.59 ± 0.055	4.40 ± 0.200	4.01 ± 0.237	3.87 ± 0.163	4.53 ± 0.138	*p* > 0.6
C17:1n-7	0.63 ± 0.043	0.90 ± 0.030	0.61 ± 0.063	0.60 ± 0.087	0.59 ± 0.093	0.73 ± 0.038	*p* > 0.05
C18:1n-9	31.79 ± 0.400	31.22 ± 0.280	31.87 ± 0.335	32.33 ± 0.212	31.90 ± 0.550	31.76 ± 0.169	*p* > 0.5
C20:1n-9	3.44 ± 0.229	3.58 ± 0.058	3.48 ± 0.078	3.31 ± 0.170	3.29 ± 0.128	3.47 ± 0.056	*p* > 0.6
C22:1n-9	0.05 ± 0.020	0.05 ± 0.013	0.05 ± 0.013	0.04 ± 0.015	0.04 ± 0.008	0.04 ± 0.005	*p* > 0.9
C24:1n-9	1.19 ± 0.020	1.25 ± 0.015	1.20 ± 0.033	1.20 ± 0.073	1.23 ± 0.038	1.24 ± 0.030	*p* > 0.8
∑MUFA ^2^	41.79 ± 0.172	41.75 ± 0.155	41.75 ± 0.450	41.91 ± 0.161	41.38 ± 0.438	41.89 ± 0.385	*p* > 0.8
C18:2n-6	16.37 ± 0.214	16.01 ± 0.055	16.11 ± 0.185	16.17 ± 0.352	16.15 ± 0.281	16.05 ± 0.236	*p* > 0.9
C18:3n-3	1.50 ± 0.087	1.59 ± 0.018	1.40 ± 0.200	1.55 ± 0.058	1.35 ± 0.038	1.55 ± 0.035	*p* > 0.5
C18:3n-6	0.13 ± 0.019	0.14 ± 0.018	0.13 ± 0.010	0.11 ± 0.009	0.12 ± 0.033	0.14 ± 0.013	*p* > 0.8
C20:2n-6	0.13 ± 0.015	0.19 ± 0.023	0.22 ± 0.018	0.12 ± 0.015	0.17 ± 0.028	0.17 ± 0.030	*p* > 0.1
C20:3n-3	0.25 ± 0.032	0.26 ± 0.070	0.30 ± 0.013	0.28 ± 0.020	0.29 ± 0.035	0.26 ± 0.023	*p* > 0.9
C20:3n-6	0.46 ± 0.037	0.42 ± 0.023	0.53 ± 0.020	0.38 ± 0.015	0.45 ± 0.025	0.48 ± 0.038	*p* > 0.06
C20:4n-6	0.94 ± 0.028	1.05 ± 0.035	1.05 ± 0.023	1.00 ± 0.025	0.93 ± 0.053	1.03 ± 0.067	*p* > 0.3
C20:5n-3	4.72 ± 0.175	4.47 ± 0.146	4.47 ± 0.078	4.47 ± 0.066	4.44 ± 0.119	4.75 ± 0.068	*p* > 0.3
C22:2n-6	0.45 ± 0.058	0.38 ± 0.008	0.40 ± 0.020	0.34 ± 0.015	0.41 ± 0.043	0.38 ± 0.023	*p* > 0.3
C22:6n-3	6.35 ± 0.099	6.50 ± 0.075	6.32 ± 0.116	6.47 ± 0.105	6.76 ± 0.084	6.41 ± 0.118	*p* > 0.1
∑n-3 HUFA ^3^	11.32 ± 0.188	11.23 ± 0.172	11.09 ± 0.189	11.21 ± 0.121	11.49 ± 0.130	11.41 ± 0.015	*p* > 0.4
Unknown	2.20 ± 0.244	1.71 ± 0.097	2.51 ± 0.214	2.86 ± 0.499	2.83 ± 0.070	2.17 ± 0.521	

^1^ ∑SFA: Total content of saturated fatty acid. ^2^ ∑MUFA: Total content of monounsaturated fatty acid. ^3^ ∑ n-3 HUFA: Total content of n-3 highly unsaturated fatty acid.

**Table 11:** Diet price (USD/kg), economic conversion ratio (ECR, USD/kg), and economic profit index (EPI, USD/fish) of the experimental diets for red sea bream.

Experimental Diets	Diet Price (USD/kg)	ECR ^1^ (USD/kg)	EPI ^2^ (USD/fish)
Con	1.74	1.52 ± 0.018 ^a^	0.78 ± 0.014 ^ab^
TBM20	1.67	1.41 ± 0.026 ^b^	0.79 ± 0.012 ^ab^
TBM40	1.59	1.33 ± 0.026 ^c^	0.80 ± 0.011 ^a^
TBM60	1.51	1.30 ± 0.009 ^c^	0.75 ± 0.005 ^bc^
TBM80	1.43	1.27 ± 0.019 ^cd^	0.74 ± 0.006 ^c^
TBM100	1.36	1.22 ± 0.016 ^d^	0.70 ± 0.015 ^d^
*p*-value		*p* < 0.0001	*p* < 0.0001

Values (means of triplicate ± SE) in the same column sharing the common superscript letter are not significantly different (*p* > 0.05). ^1^ Economic conversion ratio (ECR, USD/kg) = Feed consumption of fish (kg/fish)/weight gain of fish (kg/fish) × diet price (USD/kg). ^2^ Economic profit index (EPI, USD/fish) = [final weight of fish (kg/fish) × selling price of fish (USD/kg)] − [feed consumption of fish (kg/fish) × diet price (USD/kg)].

## Data Availability

The data are available from the corresponding author upon reasonable request.
